# Untargeted Metabolomics of Epimastigote Forms of *Trypanosoma cruzi*

**DOI:** 10.21769/BioProtoc.5368

**Published:** 2025-07-05

**Authors:** Michel Augusto Silva, Mario Izidoro, Bruno Souza Bonifácio, Sergio Schenkman

**Affiliations:** 1 Department of Microbiology, Immunology, and Parasitology, Escola Paulista de Medicina, Universidade Federal de São Paulo, São Paulo, Brazil; 2 Hospital São Paulo, São Paulo, Brazil; 3 ARIES, Antimicrobial Resistance Institute of São Paulo, São Paulo, Brazil

**Keywords:** GC-MS, *T. cruzi*, Metabolomics, Bioinformatics, Metabolite, Protozoan, Metabolism

## Abstract

*Trypanosoma cruzi*, the causative agent of Chagas disease, faces significant metabolic challenges due to fluctuating nutrient availability and oxidative stress within its insect vector. Metabolomic techniques, such as gas chromatography–mass spectrometry (GC–MS), have been widely used to study the adaptive mechanisms of the parasite. This article describes a standardized method for the untargeted metabolomics analysis of *T. cruzi* epimastigote, covering parasite cultivation, sample deproteinization with methanol, metabolite extraction, derivatization with BSTFA, and GC–MS analysis. To ensure robustness and reproducibility, statistical analysis uses univariate tests, as well as multivariate approaches such as principal component analysis (PCA) and partial least squares (PLS) regression. The protocol offers a reliable and sensitive method to study metabolic responses in *T. cruzi* under environmental stress, with low biological variability and high reproducibility.

Key features

• GC–MS was used to conduct a standardized metabolomics investigation of *Trypanosoma cruzi* epimastigote, assuring reproducibility and minimum biological variability.

• Includes sample deproteinization, metabolite extraction, and derivatization with BSTFA for accurate metabolite profiling under different biological conditions.

• Employs robust statistical approaches (PCA, PLS) to investigate differences among experimental groups and detect significant alterations in metabolism.

• Internal standards and multiple replicates ensure high sensitivity and repeatability, which is excellent for investigating metabolic processes in protozoan parasites.

## Graphical overview


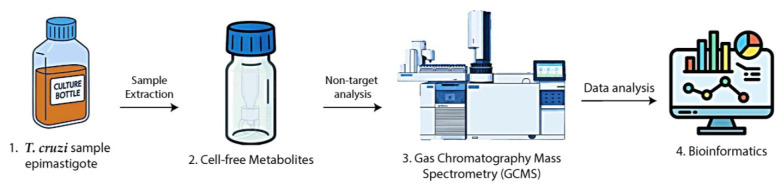


**Graphical overview.** 1. *Trypanosoma cruzi* epimastigotes are cultivated and counted. 2. Metabolite extracts are prepared, methoxylated, and silylated. 3. Samples are analyzed by GC–MS. 4. Data are processed using bioinformatics tools.

## Background

*Trypanosoma cruzi (T. cruzi*) is a flagellated protozoan and the causative agent of Chagas disease, a neglected tropical disease affecting millions worldwide [1]. The parasite undergoes a complex life cycle, alternating between an insect vector and a mammalian host, which requires metabolic flexibility to survive fluctuating environmental conditions [2]. *T. cruzi* proliferates in the insect's midgut as epimastigotes, where it encounters nutrients obtained from the mammalian host's blood through feeding, generating oxidative products from red blood cell degradation. This environment exposes the parasite to reactive oxygen species (ROS) produced by hemoglobin breakdown, demanding adaptation mechanisms to handle oxidative stress and nutritional variations [3]. Understanding how the parasite responds to these stresses is critical for identifying potential metabolic limitations that can be targeted by therapeutic approaches for Chagas disease.

Metabolomic analyses have been used to explore *T. cruzi*'s metabolic adaptations to various conditions [4]. This and other studies employed both targeted and untargeted metabolomics techniques to explore metabolic changes in *T. cruzi* during stress conditions such as dietary deprivation and oxidative stress. These findings have shed light on the parasite's adaptation mechanisms in critical circumstances [5]. However, comprehensive protocols explaining the methodology for sample preparation, metabolite extraction, and subsequent analysis utilizing gas chromatography–mass spectrometry (GC–MS) remain scarce [6,7].

We describe a standard protocol for untargeted metabolomics analysis of *T. cruzi* epimastigotes grown in normal or varied culture conditions. Our method includes a thorough workflow from parasite cultivation and sample preparation to metabolite derivatization and GC–MS analysis. This methodology identifies metabolic alterations with excellent sensitivity and reproducibility, allowing researchers to investigate *T. cruzi's* metabolic flexibility, as well as other parasites, in response to environmental stresses [8]. Compared to earlier techniques, our protocol preserves parasite viability under stress circumstances, reduces sample variability, and provides a robust bioinformatics pipeline for data analysis [9].

## Materials and reagents

**Biological materials**

1. Epimastigote forms of *Trypanosoma cruzi* DM28c strain (wild type) [10]

**Reagents**

1. Sodium chloride (Sigma-Aldrich, catalog number: S9888-1KG)

2. Potassium chloride (Sigma-Aldrich, catalog number: P3911-1KG)

3. Sodium phosphate dibasic (Sigma-Aldrich, catalog number: S9763-1KG)

4. Potassium phosphate (Sigma-Aldrich, catalog number: P0662-1KG)

5. Bacto^TM^ tryptose (BD Difco^TM^, catalog number: 226920)

6. Liver infusion broth (BD Difco^TM^, catalog number: 211713)

7. Calcium chloride (Sigma-Aldrich, catalog number: C1016-500G)

8. Magnesium chloride (MgCl_2_) (Sigma-Aldrich, catalog number: M8266-1KG)

9. Anhydrous sodium bicarbonate (Sigma-Aldrich, catalog number: 792519-1KG)

8. Hemin (Sigma-Aldrich, catalog number: H9039-1G)

9. Triethanolamine (Millipore, catalog number: 1.08379)

10. D-(+)-Glucose (Sigma-Aldrich, catalog number: G8270-1KG)

11. Penicillin G (Sigma-Aldrich, catalog number: P3032-25MU)

12. Streptomycin sulfate, *Streptomyces* sp. (Sigma-Aldrich, catalog number: 5711)

13. Fetal bovine serum (FBS) (Gibco^TM^, catalog number: 210635K)

14. Glucose oxidase from *Aspergillus niger* (Sigma-Aldrich, catalog number: G7141-50UK)

15. PIPES (Sigma-Aldrich, catalog number: P8203-50G)

16. EGTA (Sigma-Aldrich, catalog number: E3889-500G)

17. Methanol, ≥ 99.8% (GC), HPLC grade, suitable for HPLC, LiChrosolv^®^ (Supelco, catalog number: 1.06018)

18. O-methoxyamine hydrochloride [for GC derivatization, LiChropur^TM^, 97.5%–102.5% (AT)] (Supelco, catalog number: 89803)

19. Pyridine [puriss. p.a., ACS reagent, reag. Ph. Eur., ≥ 99.5% (GC)] (Sigma-Aldrich, catalog number: 33553)

20. N,O-Bis(trimethylsilyl)trifluoroacetamide (BSTFA) (for GC derivatization, LiChropur^TM^, 99.0%) (Supelco, catalog number: 15222-5ML-F) plus 1% trimethylchlorosilane (TMCS) (Pierce Chemical Co., Rockford, IL, USA)

21. Pentadecanoic acid (Sigma-Aldrich, catalog number: P6125-1G)

22. Heptane (hypergrade for LC-MS LiChrosolv^®^) (Sigma-Aldrich, catalog number: 1036541000)

23. Isopropanol [≥ 99.8% (GC), ACS reagent, reag. Ph. Eur., reag. ISO, EMSURE^®^] (Supelco, catalog number: 1096345005)

24. NaOH (Sigma-Aldrich, catalog number: 221465-1KG)

**Solutions**

1. Liver infusion tryptose (LIT) medium [11] (see Recipes)

2. Triatomine artificial urine medium (TAU) [12] (see Recipes)

3. Glucose oxidase solution (see Recipes)

4. Phosphate-buffered saline (PBS) [13] (see Recipes)

5. Hemin solution (see Recipes)

6. Methoximation solution (see Recipes)

7. Internal standard solution (IS) (see Recipes)

**Recipes**

**1. Liver infusion tryptose (LIT) medium**

Dissolve sodium chloride, potassium chloride, sodium phosphate, tryptose, and liver infusion broth in 800 mL of ultrapure water (Milli-Q), ensuring complete dissolution before adding hemin. Adjust to pH 7.2 and complete the solution volume to 1 L. Dispense the medium into 500 mL glass flasks and autoclave at 121 °C for 20 min. The medium can be stored at 4 °C for up to 3 months. Before use, supplement with glucose, fetal bovine serum, penicillin, and streptomycin. The supplemented medium should be stored at 4 °C for a maximum of 1 month. Before use, warm the medium to 28 °C, which is the optimal cultivation temperature for *T. cruzi* epimastigotes.

**Table:** 

Reagent	Weight	Volume
Sodium chloride	2 g	-
Potassium chloride	0.2 g	-
Sodium phosphate	4 g	-
Tryptose	2.5 g	-
Liver infusion broth	2.5 g	-
Hemin 10 mg/mL (see Recipe 5)	-	1 mL of 10 μg/mL stock
Glucose 20% (sterilized by filtration)	0.2%	10 mL of 0.2%
Fetal bovine serum (heat inactivated 30 min at 56 °C)	-	100 mL
Penicillin (sterilized by filtration)	-	1 mL of 59 mg/mL
Streptomycin (sterilized by filtration)	-	1 mL 133 mg/mL
Total volume	-	1,000 mL

**2. Triatomine artificial urine medium (TAU)**

Dissolve calcium chloride, magnesium chloride, anhydrous sodium bicarbonate, sodium chloride, and potassium chloride in 800 mL of ultrapure water (Milli-Q). Adjust the pH to 6.2 using a 1 M sodium phosphate stock solution and bring the final volume to 1 L. Dispense the medium into 500 mL glass flasks and sterilize the solution using a 0.22 μm filter. The medium can be stored at 4 °C for up to 3 months. Before using it, warm it to 28 °C.

**Table:** 

Reagent	Weight	Volume
Calcium chloride	0.294 g	-
Magnesium chloride	0.406 g	-
Anhydrous sodium bicarbonate	0.350 g	-
Sodium chloride	11.1 g	-
Potassium chloride	1.267 g	-
Total volume	-	1,000 mL

**3. Glucose oxidase solution**

To make the glucose oxidase (GOX) stock solution, dissolve PIPES in ultrapure water (Milli-Q) to a final concentration of 80 mM, add 1 mM EGTA and 1 mM MgCl_2_, and adjust the pH to 6.9 with NaOH in 100 mL, ensuring thorough dissolution. Then, dissolve glucose oxidase in 10 mL of this buffer to a final concentration of 6 U/mL, stirring gently to prevent enzyme denaturation. To avoid repeating the freeze-thaw cycle, divide the solution into small aliquots (500 μL) and store at -80 °C. To conduct experiments, dilute this stock solution 1:10 to a final working concentration of 0.6 U/mL in the culture medium.

**Table:** 

Reagent	Weight	Volume
PIPES	2.42 g	-
EGTA	38 mg	-
Magnesium chloride	9 mg	-
		100 mL
Glucose oxidase	60 U	-
Total	-	10 mL

**4. Phosphate-buffered saline (PBS)**

Dissolve sodium chloride, potassium chloride, sodium phosphate, and potassium phosphate in 1 L of ultrapure water (Milli-Q). Transfer the buffer to a 1 L glass flask and autoclave at 121 °C. Filter the solution through a 0.22 μm filter in a laminar airflow cabinet before aliquoting into 100 mL flasks. The sterile buffer should be kept at room temperature for a maximum of one month.

**Table:** 

Reagent	Weight	Volume
Sodium chloride	8 g	-
Potassium chloride	0.2 g	-
Sodium phosphate	1.15 g	-
Potassium phosphate	0.2 g	-
Total volume	-	1,000 mL

**5. Hemin stock solution**

Make a paste by combining 200 mg of hemin and 200 μL of triethanolamine. Dissolve the mixture in 20 mL of ultrapure water to 10 mg/mL. The solution can be kept at 4 °C for up to six months.

**Table:** 

Reagent	Weight	Volume
Hemin	200 mg	-
Triethanolamine	-	0.2 mL
Total volume	-	20 mL

**6. Methoximation solution**

Dilute O-methoxyamine hydrochloride in pyridine to 15 mg/mL and store at -20 °C.

**Table:** 

Reagent	Weight	Volume
O-methoxyamine hydrochloride	0.045 g	-
Pyridine	-	3 mL
Total volume	-	3 mL

**7. Internal standard solution**

Dilute pentadecanoic acid heptane at 1,000 ppm (1 g/L) to 10 ppm in heptane and store at -20 °C.

**Table:** 

Reagent	Weight	Volume
Pentadecanoic acid heptane	-	100 μL
Heptane	-	9.9 mL
Total volume	-	10.0 mL

**Laboratory supplies**

1. Cell culture flasks 75 cm^2^ (Biofil, catalog number: TCF001250)

2. Cell culture flasks 25 cm^2^ (Biofil, catalog number: TCF001050)

3. 15 mL graduated centrifuge tubes (Biofil, catalog number: CFT511150)

4. 50 mL graduated centrifuge tubes (Biofil, catalog number: CFT011500)

5. 5 mL serological pipettes (Biofil, catalog number: GSP010005)

6. 10 mL serological pipettes (Biofil, catalog number: GSP010010)

7. 25 mL serological pipettes (Biofil, catalog number: GSP010025)

8. 1–200 μL pipette tips (Corning, catalog number: 4711)

9. 100–1,000 μL pipette tips (Corning, catalog number: 4714)

11. Cell counting Neubauer chamber (Corning, catalog number: 480200)

12. P20 micropipette (Gilson, catalog number: F144056M)

13. P200 micropipette (Gilson, catalog number: F144058M)

14. P1000 micropipette (Gilson, catalog number: F144059M)

15. 1.5 mL microtube (Axygen, catalog number: MCT-150-A)

16. 1.5 mL cryotube (Corning, catalog number: 430659)

17. 1 L glass storage bottle (Pyrex, catalog number: CLS13951L)

18. 500 mL glass storage bottle (Pyrex, catalog number: CLS1395500)

19. 100 mL glass storage bottle (Pyrex, catalog number: CLS1395100)

20. Nitrile gloves (Supermax, catalog number: 011185)

21. 1 L vacuum filtration devices, pore 0.22 μm (Corning, catalog number: CLS430015-12EA)

## Equipment

1. Laminar airflow cabinet (Trox, catalog number: 583/FLV)

2. Tabletop refrigerated centrifuge for 15 and 50 mL tubes (Thermo Scientific, model: Sorvall^®^ Legend^TM^ Mach 1.6R)

3. Microcentrifuge refrigerated for 1.5 and 2 mL microtubes (Thermo Scientific, model: Sorvall^®^ Legend^TM^ Micro 17R)

4. Vortex mixer (Daigger, model: Vortex Genie 2, catalog number: 22220A)

5. Ultrasonic bath (Synth, model: SSBuc 6l)

6. 4 °C refrigerator (Consul, catalog number: CDR46/127)

7. -20 °C freezer (Consul, catalog number: CDR46/127)

8. Ultra-low -80 °C freezer (Panasonic, catalog number: MDF U33V-PA, VIP Series)

9. Liquid nitrogen (N_2_) storage tank (Bridgepath^®^ International Cryogenics Director D4000)

10. GC–MS chromatography coupled with a mass spectrometer quadrupole type (Shimadzu Co., model: QP2020NX)

11. SLB^TM^-5MS fused silica capillary column (30 m × 0.25 mm, 0.25 μm thickness, Supelco)

12. Helium gas 5.0 analytical grade (White Martins Ltda.)

13. Electronic balance (Bioprecisa, catalog number: FA 2104R)

14. SpeedVac concentrator (Eppendorf, model: Vacufuge^®^)

15. Fume hood (Braslab^®^ Propylene Box)

16. 28 °C BOD incubator (Tecnal, model: TE-371)

17. 70 °C incubator (SureTemp^® ^Dual Convection incubator, catalog number: Z742691-1EA)

18. Vacuum pump (Fanem^® ^Diapump^®^, model 089/CA)

19. Serological pipette controller (HTL SwiftPet^®^ Pro)

20. Racks (Prolab^®^)

## Software and datasets

1. GCMS Solution (Shimadzu Co., version 4.52) (requires a license)

2. NIST17 MASS (Shimadzu Co., version 1.00.1) (requires a license)

3. GCMS Smart Metabolite (Shimadzu Co., version 3.01) (requires a license)

4. Excel (Microsoft Office) (requires a license)

5. https://www.genome.jp/kegg/ (free to use)

6. http://www.hmdb.ca (free to use)

7. http://www.metaboanalyst.ca/ (free to use)

8. Orange 3 (free to use)

## Procedure

**A. Parasite maintenance and stress induction**

**Figure 1.**
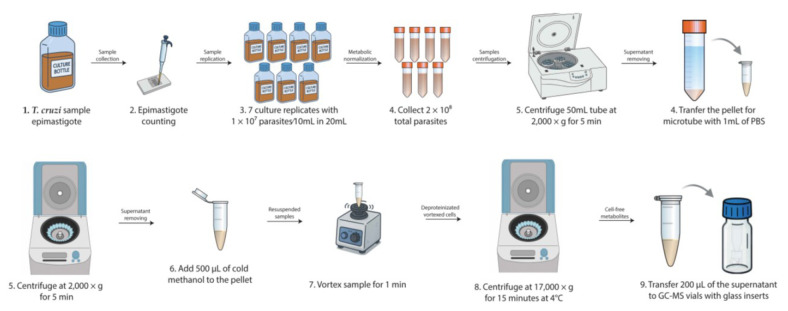
Workflow for sample collection and deproteinization (steps A1–6). This workflow outlines the entire procedure from parasite sample collection to the deproteinization and metabolite collection for GC–MS analysis.

1. Maintain *T. cruzi* epimastigotes in LIT medium in a tissue culture flask at 28 °C (25–150 cm^2^), depending on the volume used (the flasks can stay upright as epimastigotes do not attach).

2. For nutritional and oxidative stress, the parasites should be in the exponential growth phase (2 × 10^7^/mL, counted with a Neubauer chamber [14] or other counting apparatus).

3. Collect and centrifuge the required quantity of parasites at 2,000× *g* for 5 min. Then, remove the supernatant using vacuum aspiration within the flow hood and resuspend to 1 mL with PBS (Figure 1).

4. Evenly divide the parasite suspension for the controls and experimental conditions desired. Repeat centrifugation.

5. To induce nutritional stress, resuspend one tube of *T. cruzi* epimastigotes in TAU medium and the other in standard medium to a concentration of 1 × 10^7^/mL. Allocate the suspensions into 7 culture flasks, each containing 20 mL, and incubate for 3 h at 28 °C.

6. To induce oxidative stress, resuspend the parasites in LIT medium to a concentration of 1 × 10^7^/mL and allocate them into 14 culture flasks, each containing 20 mL of the parasite suspension. After 2.5 h at 28 °C, introduce 2 mL of glucose oxidase solution (GOX) to 0.6 U/mL in the 7 flasks, while adding only the GOX buffer to the remaining flasks. Incubate the cultures for 30 min at 28 °C. These approaches facilitate the production of various stress situations to examine the parasite's metabolic response, although alternative media and treatments may also be suitable.

**B. Sample preparation**

**Figure 2.**
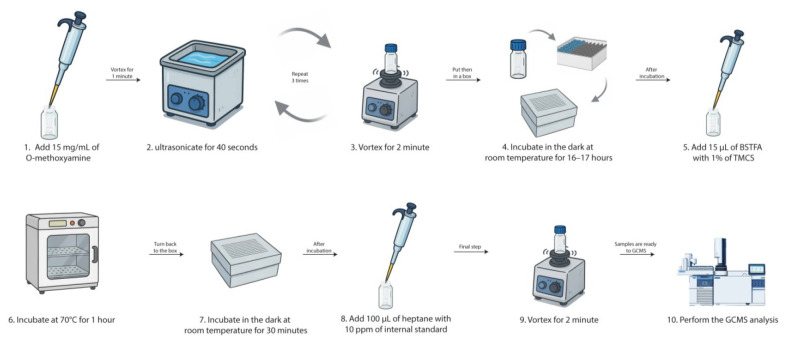
Workflow for sample methoximation and silylation (steps B1–10). This workflow outlines the entire procedure from metabolite preparation to GC–MS analysis.

1. Upon completion of the incubations, collect the total volume from each flask (2 × 10^8^ total parasites) and centrifuge at 2,000× *g* for 5 min in a 50 mL tube (Figure 2).

2. Remove the supernatants, resuspend the pellets in 1 mL of cold PBS for washing, and transfer the suspension to a microtube.

3. Centrifuge at 2,000× *g* for 5 min and carefully discard the supernatants.

4. Resuspend the parasite pellets in 500 μL of cold methanol (P.A.) to ensure complete deproteinization of each sample.

*Note: Methanol used in this step must be of high purity (e.g., hypergrade LC–MS/MS) and handled as little as possible prior to use, as exposure to ambient moisture can increase its water content, potentially interfering with the analysis. To ensure reagent quality, water content can be monitored using Karl Fischer titration [15].*

5. Collect 2 microtubes containing 300 μL of growth media and add 200 μL of cold methanol to extract the molecules present in the medium as a control, thereby minimizing analytical interference in the parasite samples.

6. Vortex the samples vigorously for 1 min, then centrifuge at 17,000× *g* for 15 min at 4 °C.

**Pause point:** If the analysis cannot be performed on the same day, the entire supernatant (500 μL) may be preserved in cryotubes within liquid nitrogen for a period of up to one week. Subsequently, return to the protocol at step B7.

7. Transfer 200 μL of the resulting supernatant from each sample to GC–MS vials containing glass inserts.

*Note: The vials do not need to be refrigerated or pre-chilled; only the methanol should be kept cold.*

8. Transfer 200 μL of each blank sample (culture medium + methanol) to GC–MS vials containing glass inserts.

9. Combine 28 μL from each of the 7 extracted samples (supernatants from the 500 μL methanol-lysed samples) to generate 3 quality control (QC) samples and transfer the resulting mixture to GC–MS vials with glass inserts. The pooled QC samples will be used for subsequent comparisons in molecular analysis and assessment of injection quality.

10. Collect 200 μL of the same cold methanol in GC–MS vials containing glass inserts to reduce methanol interference in future analyses.

11. Dry all GC–MS vials (samples, blanks, QC, and methanol control) in a SpeedVac at 30 °C.

**Pause point:** If the analysis is not performed on the same day, the dried analyte pellets can be stored at -80 °C.

*Notes:*

*1. For storage at -80 °C, GC–MS vials should be placed inside a sealed box with silica gel and enclosed in a plastic bag to minimize moisture exposure. This helps preserve sample integrity and prevents rehydration of the dried material.*

*2. For this analysis, as well as any other experiments involving parasite cultures in various media, it is imperative to utilize the identical culture medium on the same day for all repetitions. The seven replicates, regardless of being from normal or treated cultures, must be prepared on the same day to reduce variability arising from temperature fluctuations, handling discrepancies, reagent and culture medium batches, serum composition, and other experimental conditions.*

**C. Methoximation and silylation**

1. Add 15 μL of 15 mg/mL O-methoxyamine hydrochloride in pyridine to each sample.

2. Vortex for 1 min, then perform bath ultrasonication for 40 s, followed by another vortex for 2 min (3 times).

3. Incubate in the dark (inside a box) at room temperature for 16–17 h.

4. After incubation, add 15 μL of BSTFA with 1% TMCS.

*Note: BSTFA and TMCS are highly sensitive to moisture, and their degradation may compromise derivatization efficiency and GC–MS results. After opening the bottles, prompt use is recommended. Alternatively, aliquots can be prepared and stored in airtight vials placed inside a sealed container with silica gel. Aliquots may be stored at -20 °C for up to 4 weeks, protected from light and humidity to preserve reagent stability.*

5. Vortex each tube for 5 min.

6. Incubate the samples for 1 h at 70 °C for silylation.

7. Cool the samples for 30 min at room temperature in the dark.

8. Afterward, add 100 μL of 10 ppm pentadecanoic acid in heptane as an internal standard.

9. Vortex for 2 min at room temperature.

10. Proceed immediately with the GC–MS analysis.

**D. Data acquisition**

1. Samples are analyzed using a gas chromatograph coupled with a quadrupole mass spectrometer.

2. The carrier gas helium is set at a flow rate of 20 mL/min, with a flow rate of 1.36 mL/min for the column.

3. The initial temperature is set to 80 °C, with a 15 °C/min increase until 300 °C (hold for 8 min).

4. The injector, transfer line, ion source, and quadrupole are set to 280, 200, 150, and 150 °C, respectively.

5. Adjust to inject 1 μL of each sample through an automatic injector into an SLB^TM^-5MS fused silica capillary column (30 m × 0.25 mm, 0.25 μm thickness).

6. Operate in full scan mode (m/z 40–650) at 70 eV, generating three spectra per second.

7. To ensure minimal variance between injections, a pooled QC sample—prepared by mixing aliquots of all biological samples—is injected after every four biological samples. This QC helps monitor instrument performance and data consistency throughout the batch. A blank sample containing only the extraction solvent must be injected at the beginning, in the middle, and at the end of the run to assess potential contamination and identify background signals.

8. A retention time correction (TRC) method is applied to minimize retention time variability across all analyses [16].

9. For optimal sequence planning, we strongly recommend referring to the injection order table provided (see Table 1). This schedule outlines the recommended intervals for QC and blank injections to maximize data quality and enable proper quality assessment.

10. Typical chromatograms last for 34 min.

**Table 1.** Sample injection order used during GC–MS runs. Blank samples (extraction solvent only) were injected at the beginning, middle, and end of the sequence to monitor background contamination. Quality control (QC) samples—prepared by pooling aliquots from all experimental samples—were injected every four samples to evaluate data consistency and instrument stability. This injection pattern was repeated throughout the batch.

Injection #	Sample type	Description
1	Blank	Extraction solvent only
2	QC	Pooled mix of all samples
3	Biological samples	Sample 1
4	Biological samples	Sample 2
5	Biological samples	Sample 3
6	Biological samples	Sample 4
7	QC	Re-injected pooled QC
8	Biological samples	Sample 5
9	Biological samples	Sample 6
10	Biological samples	Sample 7
11	Blank	Extraction solvent only
12	QC	Re-injected pooled QC
13	-	Continue pattern

*Note: This sequence was repeated throughout the analytical batch, maintaining regular QC and blank injections to ensure system stability, detect contamination, and monitor data quality.*

**E. Data preprocessing**

**Figure 3.**
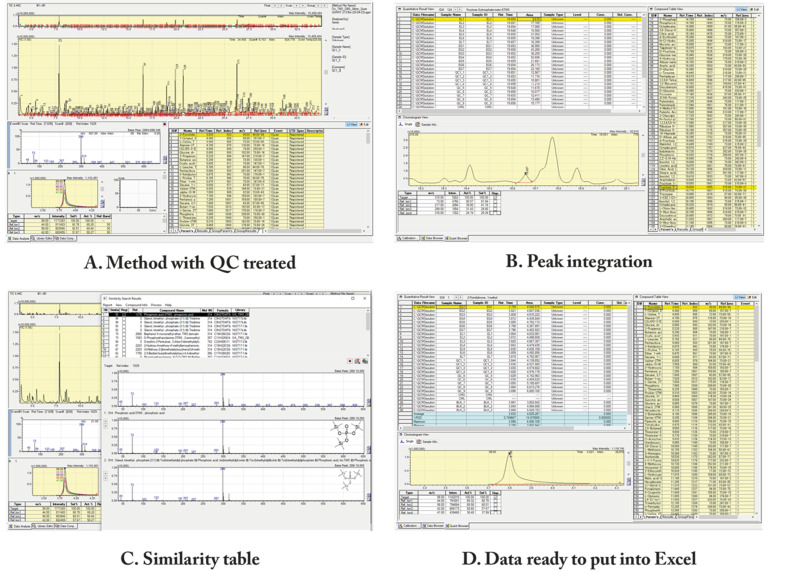
Shimadzu screens of LabSolutions^®^ browser. (A) LabSolutions^®^ with a QC treated method. (B) Peak integration, where you can see the manual delimitation of a peak. (C) The similarity table is used to see how reliable the software's identification of each metabolite is. (D) After all peak analysis and integration, the data is ready to be copied and pasted into an Excel spreadsheet (supplementary information).

1. Open the LabSolutions^®^ post-run analysis to begin the chromatographic peak integration procedure.

2. Begin by utilizing a QC chromatogram to adjust the integration parameters, such as slope, width, and smoothing (Figure 3A). Save the method as “QC treated.”

3. Use the same parameters to integrate all chromatograms to create a compound table for automatic identification utilizing Shimadzu's internal libraries.

4. Open the LabSolutions^®^ Browser software to manually correct and integrate (Figure 3B).

5. Choose the method with QC treated to be the basis for all other peak analyses.

6. Load all chromatograms and reintegrate with the same parameters as before.

a. Correct the retention time for the non-integrated peaks manually by hitting each compound in the compound table (Figure 3B).

b. For low-scoring identifications (less than 80%) with the internal library, as illustrated in Figure 3C, consider employing an external database (HMDB). This can be accomplished by copying the relevant m/z fragments and intensities and pasting them into the HMDB GC–MS search. If necessary, adjust the compound table for that analyte.

7. Save each step to avoid losing any modifications.

8. **Attention:** Even peaks found automatically by the software must be reviewed, as the machine may have erroneous recognition.

**F. Data treatment**

1. Copy all of the compound table areas, with their names and retention times (Figure 3D), into a spreadsheet labeled "raw data." Include all quality control, samples, and blanks for each metabolite.

2. Before proceeding, inspect the “raw data” spreadsheet for missing values (NA). These values must be carefully reviewed and excluded from further analysis, as they likely reflect undetected compounds or inconsistent measurements. Metabolites with NA values should not be used in the subsequent data organization and normalization steps.

*Note: Missing values were excluded from the analysis as they reflect undetected compounds or inconsistent measurements. Excluding these data is essential to ensure the accuracy and integrity of the results in the subsequent steps of data organization and normalization.*

3. Create a new sheet named “Organized Data 1” and use conditional formatting to mark all the metabolites with values equal to zero with a red color.

4. In the next sheet, put all the data labeled with a red marker (values equal to zero) and name it “Removed zero data.”

5. Copy all data from “Organized data 1” without the red-marked data, paste it into a new sheet, and name it “Organized data IS.”

6. Create a new sheet with the purpose of normalizing the numbers according to the internal standard (IS).

a. Add each metabolite in sequential rows and the respective areas of each sample in columns. Columns (A), (B), and (C) are metabolite number, retention time (RT), and name, followed by areas.

b. For example, row 1 is the column title, and row 2 is =Area2/Area$IS (metabolite area)/(internal standard area).

c. After all normalization, remove the IS row from the sheet and copy the normalized data into a new sheet, “Organized data after IS.”

7. Calculate the blank sample (BLK) normalized average for each compound and subtract it from all data.

a. Average from BLK example: =AVERAGE(BLKinitial:BLKfinal), then =AREA (metabolite data) - $(BLK AVERAGE).

b. All data with values less than zero must be removed and entered on the "Removed Data" sheet.

c. Rename the sheet to “Organized data after BLK.”

8. Copy all data from “Ordered data BLK” and paste it into another sheet. This new data is “Organized Data 2.”

a. Now, start the identification of all metabolites using the NIST data bank from the LabSolutions^®^ GC–MS data analysis.

b. All the metabolites with low similarity (<80%) must be moved to another sheet, and this sheet receives the name “Unidentified data.”

Even with modest similarity (between 70% and 80%), some metabolites are right; hence, we recommend that all metabolite instances be thoroughly examined and, if necessary, linked with RT data for improved identification correlation.

9. Copy all the remaining data from “Organized Data 2” and paste it into another sheet, “Organized Data ID.”

a. Process all metabolites by loading their compound name into HMDB (https://hmdb.ca/), PubChem (https://pubchem.ncbi.nlm.nih.gov/), ChemSpider (https://www.chemspider.com/), or any reliable software for the compound synonyms.

b. After identification, use the most common name to identify the metabolites.

c. All metabolites lacking an ID (not identified in any software) must be transferred to the “Unknown Data” sheet.

10. Copy all the remaining data from “Organized Data ID” and paste it into another sheet. This new data is “Organized Data ID OK.”

a. Identify the duplicate metabolites that came out at different retention times.

b. Sum all the values of the duplicate metabolites (example: =SUM(D3:D4)).

c. Copy all of the data from "Duplicate Data" into another sheet, removing any duplicates (just use "sum data value"). This new sheet has the name "Duplicate Data OK."

d. Then, all data with variations in QC over 30% should be removed from the table, and the resulting data should be entered into a new sheet called “Organized Data QC.” For this, use the Excel function “=(STDEV.P(QC values)/AVE(QC values)) * 100.

e. Copy all the remaining data from “Organized Data QC” and paste it into another sheet. This new data is called “Final Organized Data.”

11. Now, your data is ready to be used for statistical and functional analysis.

**G. Statistical analysis**

1. All statistical and multivariate analyses can be performed using MetaboAnalyst 6.0 ( http://www.metaboanalyst.ca/ ).

a. Data normalization is conducted using a logarithmic transformation to improve comparability among samples.

b. Univariate analyses comprise Student’s t-test for comparisons between two groups, parametric ANOVA for comparisons among several groups, and the non-parametric Kruskal–Wallis test for data that deviates from a normal distribution.

c. Multivariate analyses are applied to explore trends, detect outliers, and identify discriminant variables.

d. For each experimental condition, seven biological replicates were used, ensuring statistical robustness and reproducibility. Outlier exclusion must be done after PCA visualization and residual analysis of PLS/OPLS models, applied only when justified by technical errors or sample degradation.

**Figure 4.**
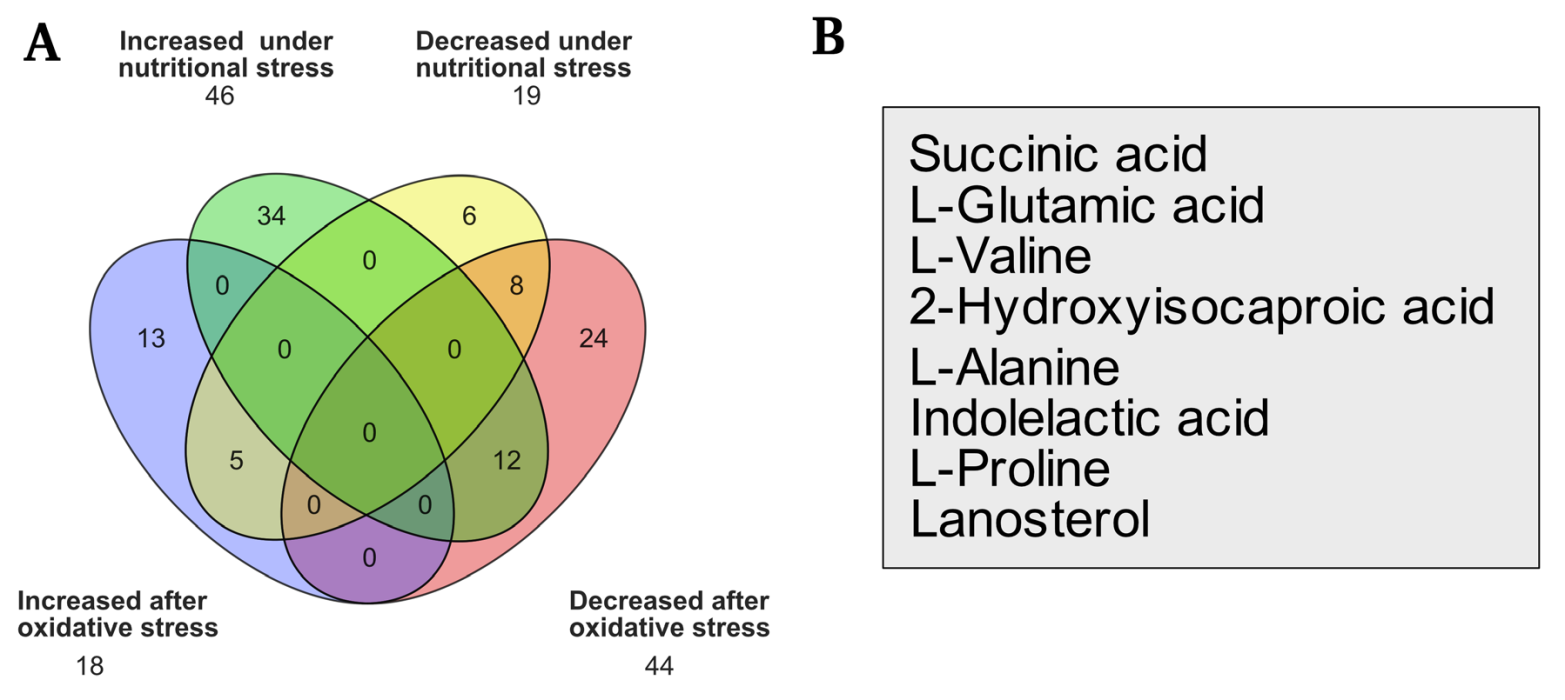
Presence and changes in several compounds under nutritional and oxidative stress conditions. (A) Venn diagram comparing increased and decreased molecules under each form of stress. (B) Molecules frequently decreased in both situations and statistical values (Silva et al. [5]).

2. Venn diagrams can be generated using the Orange 3 software [17] or any data management program to demonstrate the groups' common and distinct metabolic features. Figure 4 shows an example of the analysis, comparing the metabolites discovered under two distinct settings, as shown in Silva et al. [5]. Common metabolites that have risen or decreased can be compared (Figure 4A). Figure 4B shows the eight metabolites decreased under both nutritional and oxidative stress environments.

## Validation of protocol

**Figure 5.**
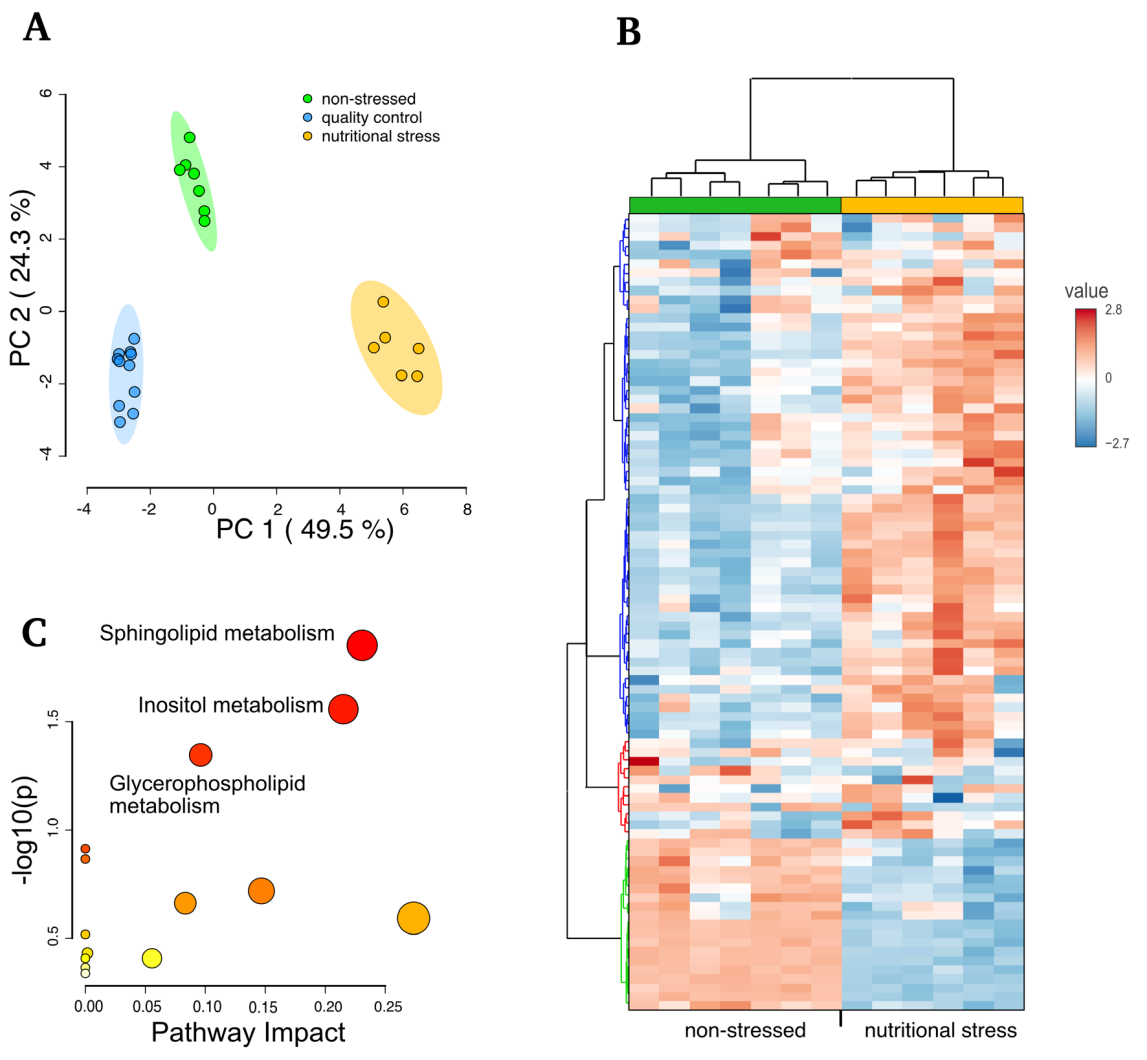
Comparative analysis of metabolites in normal growth vs. parasites incubated for 3 h in low-nutrient media (TAU). (A) PCA analysis of each sample and quality control samples. (B) Clustered heatmap of normal growing vs. stressed parasites showing the relative normalized intensities (value). (C) Analysis performed reflecting the enrichment ratio using the *T. cruzi* KEGG database in the MetaboAnalyst (adapted from Silva et al. [5]).

The method's reproducibility and robustness were validated by the variability observed in seven experimental replicates via principal component analysis (PCA). The treatment types, specifically normal growing vs. nutritionally stressed parasites, significantly cluster apart and from the quality controls (Figure 5A). The normalized intensity values were mostly consistent across each metabolite (Figure 5B), enabling the identification of the primary metabolic pathways activated by nutritional withdrawal (Figure 5C). For additional information, see Silva et al. [5].

## General notes and troubleshooting

**Troubleshooting**

**Problem 1: Excess or absence of peaks in the gas chromatogram.**

Possible cause: Excessive or low sample metabolites.

Solution: Standardize the sample volume in accordance with the device's sensitivity.

**Problem 2: Contamination.**

Possible cause: Contamination from culture media or solvent (e.g., inferior quality of methanol).

Solution: Consistently utilize fresh methanol and take care when managing the culture media.

**Problem 3: Variation in samples due to the use of different or non-standardized media.**

Possible cause: Usage of variable culture media or irregular environmental conditions during sample processing.

Solution: Utilize identical batches of culture medium and environmental parameters (temperature, chemicals) for all replicates on the same day to minimize biological variability and subsequent analytical inaccuracies.

**Problem 4: Derivatization failure.**

Possible cause: Insufficient incubation conditions or inadequate reactivity of the derivatizing agent.

Solution: Ensure that the incubation remains for a minimum of 1 h at 70 °C and that the samples are thoroughly mixed prior to derivatization.

**Problem 5: Excessive background noise or anomalous peaks in GC–MS analysis.**

Possible cause: Contamination from reagents or equipment, or instability of the carrier gas (helium).

Solution: Utilize fresh, high-quality reagents, conduct routine cleaning of the GC–MS system, and assess the quality of the carrier gas. Equipment maintenance should be performed as required.

**Problem 6: Reproducibility issues with data.**

Possible cause: Errors in sample handling or failures in device calibration.

Solution: Perform periodic equipment calibration tests and maintain uniform sample handling through standardized collection and preparation methods.

**Problem 7: Water ingress into sample inserts during storage at -80 °C.**

Possible cause: Condensation or moisture absorption by the sample inserts when stored at low temperatures.

Solution: Ensure samples are adequately sealed and maintained in airtight containers at -80 °C to avoid moisture accumulation. Let the samples reach room temperature before processing. Moisture must be avoided as it may disrupt the derivatization process, particularly prior to the methoximation step.

## Supplementary information

The following supporting information can be downloaded here:

1. Excel spreadsheet showing the data processing steps (Supplementary information).
